# Editorial: Developmental origin of diseases: a special focus on the parental contribution towards offspring’s adult health

**DOI:** 10.3389/fendo.2023.1196653

**Published:** 2023-05-15

**Authors:** Anurag Sharma, Manish Mishra, Kunal Sharan

**Affiliations:** ^1^ Division of Environmental Health and Toxicology, Nitte University Centre for Science Education and Research (NUCSER), Nitte (Deemed to Be University), Deralakatte, Mangaluru, India; ^2^ Department of Molecular and Chemical Biology, Tufts University School of Medicine, Boston, MA, United States; ^3^ Department of Molecular Nutrition, Council of Scientific & Industrial Research (CSIR)-Central Food Technological Research Institute, Mysuru, India

**Keywords:** epigenetics, maternal nutrition, developmental origin of adult health and diseases, paternal nutrition, developmental programing

The developmental origins of health and disease (DOHaD) hypothesis proposes that early life experiences can impact an individual’s health and disease risk throughout their lifespan. While much research in this field has focused on the role of maternal factors, recent studies have increasingly turned attention to how paternal factors can also influence offspring health outcomes. The special issue on “Developmental Origin of Diseases: A Special Focus on the Parental Contribution Towards Offspring’s Adult Health” in Frontiers in Endocrinology features 10 articles that examine various aspects of the complex relationship between parental factors and offspring health outcomes. The articles cover a broad range of topics, from the effects of maternal obesity and paternal diet on offspring health to the impact of parental smoking and mental health on offspring metabolic and mental health outcomes.

Several articles explore the link between maternal factors and offspring health outcomes. For instance, Sugino et al. revealed the association between maternal diet, bacterial diversity, and infant pathogenicity. In particular, this manuscript underlines that the presence of complex carbohydrates in the diet of women with gestational diabetes mellitus (GDM) leads to bacterial diversity in the infants, which could reduce the pathogenicity in the starting months of the infant’s life.

The transmission of epigenetic changes to the offspring is associated with various health issues. In this regard, Wang et al. studied the placental epigenome of GDM. The study found 256 differentially methylated positions between the control and GDM groups. Further, the study revealed the importance of 11 genes (*CYP2D7P1, PCDHB15, ERG, SIRPB1, DKK2, RAPGEF5, CACNA2D4, PCSK9, TSNARE1, CADM2, KCNAB2*) related to fetal growth.

Intrauterine growth restriction leads to health adversities later in life. In this line, Doan et al. provide an in-depth review of intrauterine growth restriction, epigenetic alteration, and its impact on fetal development. This review exhaustively covers the altered epigenetic mechanisms in different tissues such as fetal blood, umbilical cord, and placenta for small gestational age babies. Moreover, this review also discusses that intrauterine growth restriction phenotypes can be transferred in subsequent generations and possibly associated epigenetic mechanisms.

Gestational diabetes is a common condition affecting a significant proportion of women globally. Vasilakos et al. examined the levels of umbilical cord blood leptin and IL-6 in the presence of maternal diabetes or chorioamnionitis. The findings of the study suggest that maternal diabetes during pregnancy is associated with increased leptin and IL-6 levels in the cord blood, which could potentially lead to metabolic dysfunction in offspring. These results highlight the importance of managing diabetes during pregnancy to reduce the risk of future metabolic disorders in offspring.

Another study by Yang et al. confirmed that a maternal high-fat diet during the pre-weaning period impacts the offspring’s metabolic programming. The study also found impairments of brown adipose tissue thermogenesis due to beta-adrenergic signaling in the mice that consumed a high-fat diet. Bukowski et al.’s study is centered on demonstrating the role of transient receptor potential canonical channel 1 (TRPC1) on lipid homeostasis. TRPC1 is an integral protein that regulates Ca2+ ion flux across the membrane, and the study revealed that the removal of TRPC1 showed increased adiposity in the high-fat diet consumed mouse.

Birth weight is considered a fetal growth marker *in utero*, and a retrospective study by Mao et al. on 69,000 hospital deliveries found that advanced parental age is linked to small and large gestation ages in preterm and term infants, respectively. Proper liver functioning is crucial for detoxification, nutrient absorption, and metabolism. Sarli et al. investigated the impact of restricted maternal food intake on the liver proteome of the offspring using Wistar rats. The Wistar rats were divided into three groups: 1) control (normal laboratory diet), 2) offspring born with 50% restricted maternal diet (FGR group, low birth weight), and 3) offspring appropriately grown/born with 50% restricted maternal diet (non-FGR group). The study found differential expression of 451 and 751 liver proteins between control vs. FGR and non-FGR, respectively, which are involved in cholesterol biosynthesis, thyroid hormone metabolism, fatty acid beta-oxidation, and apelin liver metabolic pathways.

Nuclear receptors have a vital biological function. Zhu et al. found a mutation in NR0B1 (nuclear receptor subfamily 0 group B member 1 gene) associated with congenital adrenal hypoplasia and pubertal development failure in an adult male, while a case report by Lei et al. found a deletion of the 6p25.3p25.2 region containing 28 protein-coding genes such as *IRF4, FOXF2, TUBB2B, and FOXC1* is associated with congenital phenotypes in a neonate through chromosomal analysis.

Due to industrial and agricultural development, thousands of chemicals contaminate the different environmental compartments, such as air, food, and water, directly or indirectly impacting organism health, including humans. Hsu and Tain comprehensively reviewed the impact of toxicants on chronic kidney disease and hypertension, providing epidemiological and experimental evidence that underlines the potential renal toxicants, prenatal chemical exposure, their major sources, associated renal toxicity, and therapeutic interventions.

The articles in this special issue cover a broad range of topics, from the effects of maternal obesity and paternal diet on offspring health to the impact of parental smoking and mental health on offspring metabolic and mental health outcomes. By shedding light on the role of both maternal and paternal factors in shaping offspring health, these articles offer valuable insights into the developmental origins of health and diseases and its potential implications for clinical practice and public health policy.

## Author contributions

AS: Conceptualization; Writing - original draft. MM: Writing - review & editing. KS: Conceptualization; Writing - review & editing. All authors contributed to the article and approved the submitted version.

